# Assessment of Knowledge about Obstetric Danger Signs and Associated Factors among Pregnant Women in Debre Tabor Town, Northwest Ethiopia

**DOI:** 10.1155/2023/1475500

**Published:** 2023-03-09

**Authors:** Mestawut Mihret, Hailegebriel Wondimu

**Affiliations:** Debre Tabor Health Science College, Debre Tabor, Ethiopia

## Abstract

**Background:**

World Health Organization estimates that 800 women die from pregnancy or childbirth-related complications around the world every day. With the assumption that “every pregnancy faces risk” women should be aware of the danger signs of obstetric complications during pregnancy, delivery, and postpartum. Indications on the prevalence of obstetric danger signs and risk factors were crucial in designing programs at different levels in reducing maternal morbidity and mortality.

**Objective:**

To assess the knowledge about obstetric danger signs and associated factors among pregnant women in Debre Tabor town, Northwest Ethiopia, 2021.

**Methods:**

A community-based cross-sectional study was conducted with 295 respondents to assess knowledge about obstetrical danger signs among pregnant women in Debre Tabor town from July to September 2021. Data were collected through self-administered questionnaires. Proportional followed by simple random sampling was used to select the study participants among the pregnant women in each of the six kebeles of the town. Adjusted odds ratios at 95% confidence interval and a value of *p* < 0.05 were used to identify the predictors.

**Results:**

From a total of 295 interviewed, 61% of them were poorly knowledgeable about obstetric danger signs, but 39% of them were knowledgeable. According to our study, maternal age less than or equal to 30 years (adjusted odds ratio = 5.44; 95% confidence interval: 3.26,9.10), no formal education (adjusted odds ratio = 9.488; 95% confidence interval: 4.73, 13.14), one-time gravidity (adjusted odds ratio = 7.81; 95% confidence interval: 4.79, 9.19), and frequency of antenatal follow-up less than 4 times (adjusted odds ratio = 4.10; 95% confidence interval: 1.88, 8.96) were factors which significantly associated with the poor knowledge of obstetric danger signs.

**Conclusion:**

As the knowledge of pregnant women towards obstetric danger signs was low, maternal age less than or equal to 30 years, no formal education, one-time gravidity, and less than 4 times the frequency of antenatal follow-up are associated factors for poor knowledge on obstetric danger signs.

## 1. Background

World Health Organization (WHO) estimates that 800 women die from pregnancy or childbirth-related complications around the world every day [1]. In 1990, Ethiopia planned to reach the Millennium Development Goal (MDG) target of 267 deaths per 100,000 deliveries by the end of 2015 [2]. According to 2014 data, the maternal mortality rate (MMR) in Ethiopia was estimated to reach 420 deaths per 100,000 live births [3].

With the assumption that “every pregnancy faces risk” women should be aware of danger signs of obstetric complications during pregnancy, delivery, and postpartum [4]. The knowledge will ultimately empower them and their families to make prompt decisions to seek care from skilled birth attendants [5]. Danger signs during pregnancy include severe vaginal bleeding, swollen hands or face, blurred vision, and excessive vomiting. The commonest danger signs during childbirths are severe vaginal bleeding, prolonged labor, convulsions, and retained placenta, while danger signs during postpartum include severe vaginal bleeding, high fever, and uterine prolapse [3, 5].

A study in Nigeria revealed that 76.6% of the study participants were knowledgeable about obstetric danger signs [6]. Other studies were revealed in Southern Ethiopia, Angolela Tera district, and East Gojjam zone, where 68.4%, 37.5%, and 55.1% were knowledgeable about obstetric danger signs, respectively [7–9].

Lower results were observed from the studies done in Arba Minch, Wolaita Sodo town, Agnuak zone, Southwest Ethiopia, Bench Maji zone, and Somali region, where 24.1%, 20.0%, 16.8%, 14.6%, and 15.5% were knowledgeable about obstetric danger signs, respectively [10–14].

A study done in Tsegedie district, Ethiopia, revealed that vaginal bleeding is the most commonly mentioned as the danger sign of pregnancy 49.1%while 58.8% of respondents mentioned at least two danger signs of pregnancy, but 35.1% of respondents did not know any danger signs of pregnancy [15]. A similar study done in AletaWondo, Ethiopia, showed that 30.4%, 41.3%, and 37.7% knew at least two danger signs of pregnancy, childbirth, and postpartum period, respectively. Vaginal bleeding, 45.9%, 55%, and 59%, was the most commonly mentioned danger sign during pregnancy, childbirth, and postpartum period, respectively [16]. Different studies conducted in Ethiopia revealed that educational status, occupation, and antenatal care (ANC) utilization had a statistically significant association with knowledge about obstetric danger signs [15, 17–19].

Lack of awareness of the significance of obstetric complication symptoms is one of the reasons for the failure of women to identify and seek appropriate emergency care. Yet, little is known about the current knowledge and influencing factors in the study area. Therefore, this study was aimed at filling the gap by assessing the knowledge and determinants of danger signs among pregnant women.

## 2. Methods and Materials

### 2.1. Study Area and Period

The study was conducted in Amhara regional state, South Gondar zone, Debre Tabor town, from July 15 to September 15, 2021. The town is located in North West Ethiopia, 665 km from Addis Ababa, and 97 km far from the capital of Amhara region, Bahir Dar. The town administration has seven kebeles. According to the 2017 population projection, the total population of the town showed 96,973 [20]. Debre Tabor town has one government specialized hospital and three health centers.

### 2.2. Study Design

A community-based cross-sectional study design was conducted.

### 2.3. Source Population

All pregnant women who had been living for at least six months in Debre Tabor town.

### 2.4. Study Population

All randomly selected pregnant women who were registered by health extension workers from the kebeles of Debre Tabor town during the study period.

### 2.5. Eligibility Criteria

Pregnant women living in Debre Tabor town who were registered by health extension workers were included in the study. However, those pregnant women who were critically ill and unable to give a response were excluded from the study.

### 2.6. Sample Size Determination

The sample size was based on the assumption of the proportion of knowledge on the obstetrical danger sign report of 24.1% [10].

With 5% marginal error and 95% confidence interval of certainty (alpha (*a*) = 0.05), the actual sample size for the study is computed using one sample population proportion formula as indicated below,

(1) $$\begin{array}{c} n=\frac{{\left(Z\left(\text{a}/2\right)\right)}^{2}\text{pq}}{W^{2}}, \end{array}$$

where *n* is the sample size and *p* is the proportion of women who knew about obstetric danger signs.

*q* is the proportion of failure (1-p); Za/_2_ is the critical value 1.96, *W* is the precision (marginal error) = 0.05, *N* is the total sample size.

Thus, the sample size was

(2) $$\begin{array}{c} n=\frac{{\left(1.96\right)}^{2}\times 0.24\times 0.76}{{\left(0.05\right)}^{2}}=280\\ N=n+n\times 10\%\left(\text{contingency}\right)=280+28=308 \end{array}$$

Therefore, 308 pregnant women in Debre Tabor were enrolled in the study.

### 2.7. Sampling Procedure

The lists of pregnant mothers were obtained from the six kebele health extension offices, Debre Tabor town. Each kebele was considered as a cluster, and the number of participants to be studied was determined using a proportional-to-size technique in each cluster followed by a simple random sampling technique which was used to select the number of subjects in each cluster. There were about 387 pregnant women in each of the six kebeles. Out of which, 308 were the sample size for this study (Table 1).

### 2.8. Data Collection Tools and Techniques

Data were collected by a face-to-face interviewer administered, predesigned, and pretested questionnaire containing information pertaining to the sociodemographic profile of the participants like age, education, occupation, socioeconomic status, marital status, frequency of ANC follow-up, and number of pregnancies were utilized. Thereafter, the participants' awareness about obstetric danger signs like convulsions, headache, excessive vomiting, high fever, breathing difficulty, epigastric pain, high blood pressure, vaginal bleeding, decreased or no fetal movements, and swelling of the feet was determined. If the participant answered “yes,” it was considered as the correct response, while answers “no” or “do not know” were considered as incorrect responses. The total knowledge scores were computed, with one point given to an incorrect response and no point given to the incorrect response.

### 2.9. Data Quality Assurance

The developed questionnaire was assessed for its content validity by the clinical midwifery experts at Debre Tabor Health Science Collegewhether the questions covering all aspects of the construct being measured. The questionnaire was then pretested by 10% of samples from pregnant women in a nearby rural kebele of town. Using SPSS version 23.0, the internal consistency (reliability) of the item was tested by calculating the Chronbach alpha, and Chronbach's alpha greater than 0.7 was considered as reliable. Data collectors who were five graduating midwifery students and two supervisors were trained for 2 days on the data collection tools and techniques of the study. The principal investigator and the supervisors checked the data for completeness, and the corrective measure was taken accordingly. The collected data were checked for completeness every day.

### 2.10. Operational Definitions

#### 2.10.1. Obstetric Danger Signs

Refers to manifestations that are easily identifiable by nonclinical personnel and necessitate skilled care during the pregnancy phase. These may be during pregnancy, labor and delivery, and the postpartum period [5].

#### 2.10.2. Poor Knowledge on Obstetrical Danger Signs

This resulted from women who mention fewer than six obstetrical danger signs (two of the danger signs during each of the three periods (pregnancy, labor/childbirth, and postpartum) [11].

#### 2.10.3. Good Knowledge on Obstetrical Danger Signs

Obtained from women who mention six or more obstetrical danger signs which might be manifested during pregnancy or labor and delivery or postpartum period [11].

### 2.11. Data Analysis

Statistical analysis was done using SPSS version 23.0 statistical software. Frequency and percentage were employed to summarize the results and presented in tables and graphs. Logistic regression was carried out to identify determinants of knowledge of obstetric danger signs.

Variables with *p* < 0.25 in the bivariate analysis were selected as a candidate for multivariate logistic regression analysis to control the effect of confounders. Adjusted odds ratios (AORs) with their 95% confidence intervals (CIs) and *p* < 0.05 were considered to have a significant association between the outcome and the independent variables.

### 2.12. Ethical Consideration

Ethical clearance was obtained from Debre Tabor University, College of Medicine and Health Science Institutional Review Board and submitted to Debre Tabor town administration health office. A cooperation letter was obtained from the town administration health office and submitted to each kebele and then forwarded to health extension workers of their kebele. Verbal informed consent was obtained from respondents. The purpose of the study was explained to the respondents before data collection. All information gained during data collection was kept confidential. After the completion of the data collection, a briefing on obstetrical danger signs was entertained.

## 3. Results

### 3.1. Sociodemographic Characteristics

Out of the total of 308 pregnant women who were planned for the study, 295 were successfully interviewed yielding a response rate of 95.78% of which 79 (26.78.22%) were in the age group of more than 30 years old; 292, married previously (56.16%), were married at the age group of 20 to 24 years; 112 (37.97%) had no formal educations. Among the study participants, 35 (11.86%) responded as their family monthly income was less than 1000 Ethiopian Birr, and 133 (45.08%) participants mentioned as greater than 15 minutes were required to reach the nearest health institution (Table 2).

### 3.2. Obstetric Characteristics

Around one-third of the respondents had two and above alive children. From the respondents, 4 (1.36%) were in the age group below 15 years during their first pregnancy. In the majority of the study subjects, 242 (82.03%) utilized family planning in their lives. Regarding gravidity and parity, 177 (60%) and 102 (34.58%) had two up to four gravidities and parities, respectively. Among the respondents, 52 (17.62%) had less than four ANC follow-ups for current pregnancy, while 243 (82.37%) of the respondents had four or more ANC follow-ups. Out of 201 who gave birth previously, 22 (11%) were delivered at home (Table 3).

### 3.3. Source of Information for Knowledge of Obstetric Danger Signs

Of the respondents, 20 (8.47%) were not advised/counseled on the danger signs that occurred during pregnancy, labor and delivery, and postpartum periods during ANC visits, but 269 (91.19%) were heard about the obstetric danger signs from health institutions (Table 4).

### 3.4. General Knowledge on Obstetrics Danger Signs

**Figure 1:**
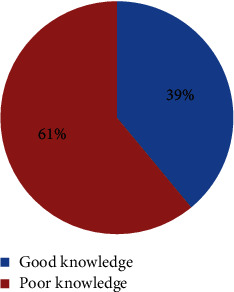
Knowledge towards obstetric danger signs among pregnant women in Debre Tabor town, North West Ethiopia, 2021.

From all of the 295 respondents, about 116 (39%) pregnant women have had good knowledge on obstetrical danger signs who mentioned at least six obstetric danger signs, whereas 179 (61%) respondents have had poor knowledge of which 160 (54%) mentioned less than six obstetric danger signs, and 19 (7%) cannot mention at least one obstetric danger sign (Figure 1).

### 3.5. Knowledge of Obstetric Danger Signs during Pregnancy

During pregnancy, vaginal bleeding 226 (76.61%), severe headache 137 (46.44%), and reduced/absence of fetal movement 89 (30.17%) were the three frequently mentioned danger signs (Table 5). From the respondents, 280 (94.92%) mentioned at least one danger sign during pregnancy.

### 3.6. Knowledge of Obstetric Danger Signs during Labor and Delivery

Severe vaginal bleeding 187 (63.39%), blurred vision 73 (24.74%), and convulsions 67 (22.71%) were the three frequently mentioned danger signs during labor and delivery (Table 5). About 241 (81.69%) of the respondents mentioned at least one danger sign during labor and delivery.

### 3.7. Knowledge of Obstetric Danger Signs during Postpartum

Severe vaginal bleeding 146 (49.49%), foul-smelling vaginal discharge 53 (17.97%), and high fever 48 (16.27%) were the three frequently mentioned danger signs during the postpartum period (Table 5). About 195 (66.10%) of the respondents mentioned at least one danger sign during the postpartum period.

### 3.8. Associated Factors with Knowledge on Obstetric Danger Signs of Pregnant Women

From all variables, maternal age, educational level, occupation, parity, and frequency of ANC follow-up for current pregnancy have shown to have an association with knowledge of obstetric danger signs. In this instance, those mothers aged greater than 30 years were 5.4 times more likely to be knowledgeable about pregnancy danger signs than mothers less than 30 years (AOR: 5.44 (3.26, 9.10)). Mothers with secondary and above were 2.3 times (AOR: 2.3 (1.4, 3.8)) and 9.4 times (AOR: 9.488 (4.73, 13.14)) more likely to be knowledgeable than mothers with primary level and with no formal educations, respectively, about pregnancy danger signs. Mothers with two and above were 7.8 times more likely to be knowledgeable than those with one gravidity (AOR: 7.8 (4.79, 9.19)). Mothers with four and above frequencies of ANC follow-up were 4.1 times more likely to be knowledgeable than mothers with less than four (AOR: 4.10 (1.88, 8.96)) (Table 6).

## 4. Discussion

The findings of this study indicated that 39% of the respondents have good knowledge on obstetric danger signs. This result is in line with studies in Angolela Tera district 37.5% [8] and East Gojjam zone 55.1% [9]. This could be due to the similarity in a study design as community-based cross-sectional was utilized in all of the three studies. The other reason might be due to the similarity in socioeconomic characteristics of the study participants as found in the same region. But the finding was lower than the studies conducted in Southern Ethiopia (68.4%) and Nigeria (76.6%) [6, 7]. The lower level of knowledge of women about obstetric danger signs could be due to the difference in the study setting, as the studies with higher results were institution based, so study participants may have more information related to healthcare than participants in community-based studies.

However, the finding is higher than the study conducted in Arba Minch, Wolaita Sodo town, Agnuak zone, Southwest Ethiopia, Bench Maji zone, and Somali region, where 24.1%, 20.0%, 16.8%, 14.6%, and 15.5% were knowledgeable about obstetric danger signs, respectively [10–14]. This might be due to a difference in sociodemographic, cultural, and health-seeking behavior of the study participants as the study areas are geographically far from each other.

In this study, maternal age, gravidity, and frequency of ANC follow-up were significant factors for pregnancy danger signs among the study participants. These findings were supported by studies conducted in Tanzania, Arba Minch, Goba, and Tsegedie district [10, 15, 19, 21]. In relation to maternal age, those mothers aged greater than 30 years were 5.4 times more likely to be knowledgeable about pregnancy danger signs than mothers less than 30 years (AOR: 5.44 (3.26, 9.10)). This could be because as the women's age increases, they can be familiar with different danger signs and get information from their colleagues.

Considering education level, mothers with secondary and above were 2.3 times (AOR: 2.3 (1.4, 3.8)) and 9.4 times (AOR: 9.488 (4.73, 13.14)) more likely to be knowledgeable than mothers with primary level and with no formal educations, respectively, about pregnancy danger signs. This might be due to educated women who may have a better understanding of danger signs by reading and listening to health-related information from different sources. Regarding gravidity, mothers with two and above were 7.8 times more likely to be knowledgeable than those with one gravidity (AOR: 7.8 (4.79, 9.19)). On the other hand, mothers with four and above frequencies of ANC follow-up were 4.1 times more likely to be knowledgeable than mothers with less than four (AOR: 4.10 (1.88, 8.96)). This might be due to having experience from their previous pregnancy and childbirth and might get information regarding pregnancy danger signs by healthcare providers.

## 5. Conclusion

This study has shown that there is a low level of knowledge on obstetric danger signs during pregnancy, childbirth, and postpartum period among pregnant women in Debre Tabor town. The most frequently mentioned danger sign during pregnancy, childbirth, and postpartum was vaginal bleeding. Hence, every woman who came to the health institution for ANC follow-up should be counseled for obstetrical danger signs in all three phases, and kebele health extension workers need to arrange the pregnant women peer groups to learn each other about obstetric danger signs. This study can be used as a base for further investigation.

## Figures and Tables

**Table 1:** The total number of pregnant women and study participants in kebele of Debre Tabor town, Northwest Ethiopia, 2021.

Kebele	Number of registered pregnant women (*n*)	Number of study participants selected by proportion (*N*) = *n* × 308/387
Kebele 1	90	71
Kebele 2	57	46
Kebele 3	67	54
Kebele 4	74	59
Kebele 5	43	34
Kebele 6	56	44
Total	387	308

**Table 2:** Sociodemographic characteristics of the respondents in Debre Tabor town, Northwest Ethiopia, 2021.

Variables	Category	Frequency (*n*)	Percent (%)
Age in years	≤30	216	73.22
	>30	79	26.78

Age at first marriage (in years)	<15	10	3.42
	15-19	88	30.14
	20-24	161	55.14
	≥25	33	11.30

Educational status	No formal educations	112	37.97
	Primary level	70	23.73
	Secondary and above	113	38 .30

Occupation	Government employee	46	15.60
	Private employee	15	5.08
	Housewife	138	46.78
	Merchant	71	24.06
	Labor worker	25	8.48

Marital status	Single	3	1.02
	Married	276	93.56
	Separated	8	2.71
	Divorced	8	2.71

Husband's educational status	No formal educations	62	7.75
	Primary level	61	14.08
	Secondary and above	161	55. 69

Have television or radio	Yes	264	89.49
	No	31	10.51

Total family monthly income	Below 1000	35	11.86
	1001-2000	87	29.49
	2001-3000	76	25.76
	Above 3000	97	32.88

Time taken to the nearby health facilities	≤15 minutes	161	54.92
	>15 minutes	133	45.08

**Table 3:** Obstetric history of pregnant women in DebreTabor town, Northwest Ethiopia, 2021.

Variables	Category	Frequency (*n*)	Percent (%)
Age at first pregnancy	<15	4	1.36
	15-19	73	24.75
	20-24	144	48.81
	≥25	74	25.08

Family planning utilization	Used	242	82.03
	Not used	53	17.97

Gravidity	One	171	57.97
	Two and above	124	42.03

Parity	Primigravida	87	29.49
	One	84	28.47
	Two-four	109	36.95
	Five and above	15	5.08

Number of ANC visits during last pregnancy	<4	56	18.99
	≥4	249	84.41

Decision maker for place delivery	Herself	207	70.17
	Husband	12	4.07
	Family member	75	25.40
	Other	1	0.36

Home delivery	Yes	22	11
	No	179	89

**Table 4:** Source of information on pregnant women about obstetric danger signs in Debre Tabor town, Northwest Ethiopia, 2021.

Sources of information	Type of information		Frequency (*n*)	Percent (%)
Counseling during ANC visit	Where to deliver	Yes	279	94.58
		No	16	5.42
	Benefit of delivering at health facility	Yes	262	88.81
		No	33	11.19
	Danger signs during pregnancy, labor and delivery, and postpartum	Yes	275	93.22
		No	20	8.78

Other sources of information	Television		45	15.25
	Radio		5	1.69
	Health facilities		269	91.19
	Other		2	0.67

**Table 5:** Knowledge on obstetric danger signs among pregnant women in Debre Tabor town, Northwest Ethiopia, 2021.

Danger signs	Knowledge during		
	Pregnancy	Labor and delivery	Postpartum
	Frequency *n* (%)	Frequency *n* (%)	Frequency *n* (%)
Vaginal bleeding	226 (76.61)	187 (63.39)	146 (49.49)
Convulsion	79 (26.78)	67 (22.71)	36 (12.20)
Severe abdominal cramp	77 (26.10)	Na	Na
Severe headache	137 (46.44)	47 (15.93)	33 (11.18)
Reduced/absence of fetal movement	89 (30.17)	Na	Na
Regular contraction before 37 weeks of gestation	25 (8.47)	Na	Na
Swelling of legs, hands, and face	75 (25.42)	55 (18.64)	40 (13.56)
Blurred vision	69 (23.39)	73 (24.74)	39 (13.22)
Labor lasting greater than 12 hours	Na	31 (10.51)	Na
Malpresentation	Na	40 (13.56)	Na
Retained placenta	Na	29 (9.83)	Na
Foul-smelling vaginal discharge	Na	Na	53 (17.97)
High fever	Na	Na	48 (16.27)
Uterine prolapsed	Na	Na	27 (9.15)
Do not know	20 (6.78)	51 (17.29)	100 (33.90)

Na = not assessed during that period.

**Table 6:** Factors having an association with pregnant women's knowledge on obstetric danger signs in Debre Tabor town, Northwest Ethiopia, 2021.

Variables	Category	Good knowledge *n* (%)	Poor knowledge *n* (%)	COR (95% CI)	AOR (95% CI)
Age	≤30	75 (64.65)	141 (78.77)	2.43 (1.45,3.50)	5.44 (3.26,9.10)^∗^
	>30	41 (35.35)	38 (21.23)	1.0	1.0

Educational level	No formal education	24 (21.62)	88 (50.57)	8.17 (4.47, 14.9)	9.488 (4.73,13.14)^∗∗^
	Primary level	19 (17.12)	51 (29.31)	5.98 (3.08, 11.58)	2.3 (1.4, 3.8)^∗∗^
	Secondary and above	78 (70.27)	35 (20.12)	1.0	1.0

Gravidity	One	56 (48.27)	115 (64.35)	3.1 (3.1,7.85)	7.81 (4.79,9.19) ^∗∗^
	Two and above	60 (51.72)	64 (35.76)	1.0	1.0

Frequency of ANC follow-up	<4	11 (6.83)	41 (30.59)	6.01 (2.94, 12.27)	4.10 (1.88,8.96)^∗∗^
	≥4	150 (93.17)	93 (69.41)		1.0

^∗∗^ *p* value of less than 0.001 and ^∗^*p* value < 0.05.

## Data Availability

The data used to support the findings of this study are available from the corresponding author upon request.

## Abbreviations

ANC: Antenatal care
HIV/AIDS: Human immune deficiency virus/acquired immune deficiency syndrome
MMR: Maternal mortality rate
WHO: World Health Organization
MDG: Millennium development goal.
